# Response Mechanism of Endogenous Hormones of Potential Storage Root to Phosphorus and Its Relationship With Yield and Appearance Quality of Sweetpotato

**DOI:** 10.3389/fpls.2022.872422

**Published:** 2022-05-23

**Authors:** Cheng-cheng Si, Qing-gan Liang, Hong-Juan Liu, Ning Wang, Sunjeet Kumar, Yan-li Chen, Guo-peng Zhu

**Affiliations:** ^1^Key Laboratory of Quality Regulation of Tropical Horticultural Crop in Hainan Province, School of Horticulture, Hainan University, Haikou, China; ^2^State Key Laboratory of Crop Biology, Agricultural College, Shandong Agricultural University, Tai’an, China

**Keywords:** sweetpotato, endogenous hormone, storage root, yield, appearance quality

## Abstract

Field and pot experiments were conducted to explore the response mechanism of endogenous hormones of potential storage root to phosphorus and its relationship with yield and appearance quality of sweetpotato using five different rates of phosphorus addition. Application of adequate amounts of phosphorus (P_2_ treatment, 112 kg of P_2_O_5_ ha^–1^ in field experiment or 0.04 g of P_2_O_5_ kg^–1^ in pot experiment) improved the yield and the appearance quality of sweetpotato when compared to the control treatment. This observation can be attributed to the fact that P_2_ treatment significantly increased the expression of *Ibkn1* and *APRT* genes and the concentration of ZR from 20 to 40 days after planting, but the results were the opposite at 10 days after planting. In addition, an increase in the expression of *SRD1*, *NIT4*, *IbMADS1*, and *OPR3* and the concentrations of IAA and JA from day 10 to day 40 after planting were observed. Furthermore, the expression of *GA3oX4* and the concentration of GA_3_ decreased significantly from 20 to 30 days of planting and significantly increased after 40 days of planting. Moreover, a significant decrease in the expression of *AAO* and concentration of ABA was observed from 10 to 30 days after planting, and a significant increase was observed after 40 days of planting. The results show that P_2_ treatment promoted root development, particularly significantly increased the number of roots and potential storage roots. P_2_ treatment significantly increased the diameter, weight, and number of storage roots at 40 days after planting. Finally, proper phosphorus application (112 kg of P_2_O_5_ ha^–1^) increased the yield (enhanced from 18.99 to 25.93%) by increasing the number of storage roots per plant and improving the appearance quality by increasing the length/diameter ratio and uniformity of storage root weight.

## Introduction

Sweetpotato (*Ipomoea batatas* L.) is an important food crop in the world. The content of phosphorus (P) in many soils of the world is often low or occurs in fixed forms (Yang et al., 2014; Roy et al., 2016). P is an essential nutrient for plant growth and development, as it affects P uptake and endogenous hormone metabolism (Shen et al., 2012; Ding et al., 2021; Zhao et al., 2021), and its deficiency dramatically affects the productivity of crops, such as sweetpotatoes, potatoes, chillies, soybean, rice, and so on (Sun et al., 2019: Kareem et al., 2020; He et al., 2021; Khanal et al., 2021; Nyiraneza et al., 2021; Yang et al., 2022). Previous studies have focused on the role of P in its uptake, yield, shape of the storage root, starch content, and total sugar quality of sweetpotato (El-Deen et al., 2011; Wang et al., 2017; Villordon et al., 2018; Munda et al., 2019; Kareem et al., 2020). These mentioned studies reported that under the conditions of a certain cultivation density, the yield is determined both by the average number of storage roots per plant and storage root weight, and between these two factors, the average number of storage roots per plant makes a greater contribution to the yield (Si et al., 2018). These traits are closely linked to root growth and development, as well as to the differentiation and formation of storage roots (Villordon and Clark, 2014).

In sweetpotatoes, initiation (0–20 days after planting) and formation (0–40 days after planting) of storage roots are a consequence of the synergistic interaction between several hormones (Tanaka et al., 2008; Ravi et al., 2009; Noh et al., 2010; Villordon et al., 2013; Si et al., 2018; Singh et al., 2019). The level of endogenous ZR in the storage roots of sweetpotato is regulated by the *KNOXI* gene, while *Ibkn1* and *Ibkn2* are *KNOXI* genes that are expressed in sweetpotato storage root, and their expression level in the initial storage root is more than two times higher than that observed in the fibrous root (Tanaka et al., 2008; Firon et al., 2013). Noh et al. (2010) found that when exogenous IAA was applied to the adventitious roots of the sweetpotato, the expression level of the *SRD1* gene and the concentration of endogenous IAA in roots increased synchronously, and its expression level in the storage roots was much higher than that of fibrous roots. Bae et al. (2011) found that the overexpression of *SRD1* in transgenic sweetpotato resulted in the production of double the number of storage roots. Studies by Ku (2008) showed that under *in vitro* culture conditions, jasmonic acid (JA) (10–20 μM) combined with 6-benzylaminopurine (BA) (10 μM) could induce the expression of *IbMADS1*, whereas application of BA or JA alone was not effective. Furthermore, *IbMADS1* was expressed mainly in the early growth stage of storage root; however, no transcript was detected in any of the examined tissues in storage root-deficient *Ipomoea* species, such as *I. leucantha* and *I. trifida* (Ku, 2008). Previously, Singh et al. (2019) reported that exogenous application of GA_3_ upregulated the expression of vascular development regulatory genes and lignin biosynthesis genes, and downregulated the expression levels of starch biosynthesis genes in the young roots, which consequently delayed the storage root formation and reduced the number of storage roots (Singh et al., 2019). Most studies suggested that ABA mainly regulates the bulking and development of storage roots, but its effect is not observed in the storage root formation of sweetpotato (Wang et al., 2006; Zhang et al., 2020). Furthermore, adenine phosphoribosyltransferase (APRT), regulated by the gene *APRT*, participates in the activation of cytokinin biosynthesis in plants (Mok and Mok, 2001; Auer, 2002; Zhou et al., 2006; Wu et al., 2008). *Nitrilase 4 (NIT4)* exhibits nitrilase activity and catalyzes the conversion from indole acetonitrile to IAA (Piotrowski et al., 2001; Wang et al., 2019). 12-oxo-10,15(Z)-phytodienoic acid reductase 3 (OPR3) is a key enzyme in JA synthesis (Schaller and Stintzi, 2009; Wasternack and Hause, 2013), and is regulated by the gene *OPR3*. The abscisic aldehyde oxidase (AAO) is controlled by the gene *AAO* that converts xanthoxin into ABA in the process of ABA biosynthesis (Chen et al., 2020). GA3-oxidase (GA3ox) is a key enzyme in gibberellin (GA) biosynthesis (Li and Li, 2019).

Overall, the effect of phosphorus on endogenous hormones in the potential storage root and the storage root formation is not clearly explored. According to the studies of Wang et al. (2015) and Jia et al. (2016), proper phosphorus application (112.5 kg of P_2_O_5_ ha^–1^) can improve the P uptake, phosphorus utilization efficiency, and yield, while excessive phosphorus application will reduce the phosphorus utilization efficiency. Therefore, P treatment rates of 0, 56, 112, 168, and 224 kg of P_2_O_5_ ha^–1^ were followed in the current study, and the response mechanism of endogenous hormones of potential storage root to phosphorus and its relationship with yield and appearance quality of sweetpotato was investigated.

## Materials and Methods

### Materials

Two widely cultivated orange-fleshed sweetpotato cultivars of China, Yanshu25 (YS-25) and Pushu32 (PS-32), with about four storage roots were used for this experiment. The young and healthy vegetative terminal cuttings grown from the storage roots having the following characteristics were used: length of about 25 cm with excess buds and leaves removed and the top three fully unfolded leaves retained. The cutting base was soaked in 30 mg/kg carbendazim for 5 min. After drying, the cuttings were planted into four nodes by the oblique planting method. In this experiment, the following fertilizers were used: urea (N, 46%; Sinopec, Co., Ltd., China), calcium superphosphate (P_2_O_5_, 16% and S, 8%; SDIC Xinjiang Lop Nur Potassium Salt Co., Ltd., China), and potassium sulfate (K_2_O, 52% and S, 17.5%; Guangdong Zhanhua Group Co., Ltd., China). The micronutrient fertilizer (Shenzhen ORTIES Biotechnology Co., Ltd., China) with a nutrient content of Mg ≥ 20%, Zn ≥ 4%, H_3_BO_3_ ≥ 7%, Fe ≥ 4%, Mo ≥ 0.8%, Ca ≥ 6%, Cu ≥ 0.5%, Mn ≥ 0.8%, Se ≥ 0.4%, SiO_2_ ≥ 2%, I ≥ 0.3%, Sn ≥ 0.5%, Sr ≥ 0.2%, Br_2_ ≥ 0.3%, yellow humic acid ≥ 20%, and microorganism composite ≥ 5% was applied. The specification of the pot was 20 cm in height, 31 cm upper diameter, and 28 cm lower diameter.

### Experimental Design

The experiment was conducted at the agricultural base of Hainan University, Haikou, China (20°06′ N, 110°33′ E). The first pot experiment was carried out from 4 November 2019 to 14 December 2019, and the second one was carried out from 16 November 2020 to 26 December 2020. The first field experiment was carried out from 28 November 2020 to 2 March 2021, and the second one was carried out from 1 June 2021 to 30 September 2021. Two-factorial pot experiments were carried out in triplicates. Different concentrations of base fertilizer (0, 0.02, 0.04, 0.06, and 0.08 g of P_2_O_5_ kg^–1^) were applied and represented as P_0_, P_1_, P_2_, P_3_, and P_4_, respectively. At the same time, 0.45 g of N per plot, 0.85 g of K_2_O per plot, and micronutrient fertilizer of 0.4 g per plot were applied as base fertilizer to meet the requirements of plants. About 10 kg of washed river sand was loaded into the pot, and the fertilizer and sand were mixed evenly. Field experiments were carried out to verify the results of pot experiments. The field soil (depth 0 to 0.30 m) was sandy loam with a sand content of 50.4%, soil bulk density of 1.41 g cm^3^, pH (water: soil, 1:1) of 6.84, organic matter of 1.21%, alkali-hydrolysable nitrogen of 52.56 mg kg^–1^, available phosphorus of 9.01 mg kg^–1^, available potassium of 80.63 mg kg^–1^, and exchangeable calcium of 106.34 mg kg^–1^. The field experiment was carried out as a two-factor split-plot design with triplicates in a randomized block arrangement. Two cultivars, Yan-25 and Pu-32, were assigned to the main plots, and different P_2_O_5_ treatments were assigned to sub-plots, which contained 0, 56, 112, 168, and 224 kg of P_2_O_5_ ha^–1^, also expressed as P_0_, P_1_, P_2_, P_3_, and P_4_, respectively. The amount of P_2_O_5_ applied was the same as that used in the pot experiment, which was converted according to the soil bulk density. Simultaneously, 120 kg of N ha^–1^ and 240 kg of K_2_O ha^–1^ were applied, and all the fertilizers used were base fertilizers and mixed with the soil. The distance between plants was 20 cm, the distance between ridges was 80 cm, and the plot area was 8 m^2^. The base of cuttings was soaked in 30 mg kg^–1^ of carbendazim for 5 min, and after drying, the cuttings were planted in the soil. Both field and pot experiments were carried out in the open air. Climatic growth conditions and irrigation practices followed for the cultivation of sweetpotato are detailed in Table 1.

**TABLE 1:** Climatic growth conditions for sweetpotato.

Year	Month	Temperature (°F)			Precipitation (mm)	Field irrigation (mm)
		Max	Average	Min		
2019	11	81.65	74.50	67.85	10.80	
	12	77.11	70.09	64.37	6.60	
2020	11	79.00	74.13	69.58	14.80	100
	12	70.87	66.33	62.67	6.20	100
2021	01	68.39	61.44	55.35	19.10	
	02	76.70	67.67	60.40	26.20	
	03	83.65	75.57	70.19	2.00	
	06	94.27	84.79	78.97	88.50	100
	07	92.44	84.39	78.22	133.80	
	08	92.10	83.30	77.33	172.70	
	09	89.67	81.62	75.83	83.40	

### Sampling and Measurements

#### Agronomic Traits

In the pot experiment, the roots were washed with water on days 10, 20, 30, and 40 after planting to obtain whole roots. The root number was counted and weighed manually. Afterward, a root scanner (EPSON EXPRESSION 10000XL, China) was used to scan the whole root system. Similarly, the WinRHIZO 2009 root analytical procedure was used to analyze the scanned root system images to obtain the data regarding root length, average root diameter, root surface area, root volume, and root tip number. Six thickest roots of each plant were selected from the whole root system as potential storage roots. The diameter of the thickest part was measured using a Vernier caliper, and the fresh weight was calculated by using a weighing balance. After planting for 40 days, the division method of Si et al. (2018) was used to calculate the number of potential storage roots per plant (diameter, 2.00 mm) and weighed.

In the field experiment, 40 days after planting (canopy closure), five representative plants were selected, and the potential storage root number and potential storage root weight per plant were investigated. After planting for 120 days (harvest time), all the storage roots were dug out, and then the number and weight of the storage roots were determined. After that, the average storage root number per plant, average storage root weight, and yield were calculated. Five representative plants per plot were selected to measure the diameter and the length of storage root, and then the length/diameter ratio (L/D ratio) was calculated according to the protocol of Villordon and Carroll (2002) and Villordon et al. (2013). L/D ratio was used to describe the shape of agricultural products (Costa et al., 2011; Villordon et al., 2018), where if an object has an L/D ratio = 1, then it is considered to have a circular shape (Kaiser et al., 2006). The uniformity of storage root weight is expressed by the coefficient of variation (CV) (CV = standard deviation of storage root weight/average of storage root weight). The smaller the CV, the better the uniformity of storage root weight.

#### Endogenous Hormone Concentration

For the determination of endogenous hormone concentration (Si et al., 2018), fresh potential storage roots (1 g) were extracted, and the extract was purified by passing through C18-Sep-Pak cartridges. IAA, ZR, ABA, GA, and JA-Me concentrations were assayed using enzyme-linked immunosorbent assay (ELISA) kits with monoclonal antibodies. The mouse monoclonal antigens and antibodies against IAA, ZR, ABA, GA, and JA-Me were produced at the Hormones Research Institute (China Agricultural University). ELISA reactions were conducted in 96-well microtitration plates; each well of a plate was coated with 50 μl of sample and 50 μl of antigens (0.25 mg ml^–1^) against the hormones. The plate was incubated for 30 min at 37°C. After washing for four times with phosphate-buffered saline + Tween 20 (0.1% [v/v]) buffer (pH 7.4), 100 μl of antibodies (20 mg ml^–1^) was added to each well, and the plates were incubated for a further 30 min at 37°C. The plates were rinsed four times with phosphate-buffered saline + Tween 20 buffer, and 100 ml of color development solution [containing 2 mg ml^–1^ O-phenylenediamine and 0.004% (v/v) H_2_O_2_] was added to each well. The reaction was stopped by adding 50 ml of 2 M H_2_SO_4_ per well, that is, when the standard solution containing 2,000 ng ml^–1^ showed a pale color. Color development in each well was detected using the microplate photometer (Thermo Fischer Scientific, Multiskan FC, United States) at optical density (A490).

#### RNA Extraction, cDNA Synthesis, and Real-Time Quantitative PCR Performance

The total RNA from the potential storage root was extracted using a Plant Total RNA Isolation Kit Plus (Foregene, RE-05024, Chengdu, China). The concentration of total RNA was measured by a NanoDrop lite spectrophotometer (Thermo Fischer Scientific, Waltham, MA, United States).

First-strand cDNA was synthesized from 1 μg of DNA-free RNA using MonScript*™* RTIII All-in-One Mix with dsDNase (Monad, MR05101, Wuhan, China).

The reaction, performed in a real-time PCR machine (qTOWER3 G, Jena, Germany), was initiated with a preliminary step of 30 s at 95°C, followed by 40 cycles at 95°C for 15 s and 60°C for 30 s. Templateless controls (5.5 μl of ddH_2_O) for each primer pair were necessary for each run. The primers used in qRT-PCR are listed in Table 2, and β-actin was used as a reference gene (Si et al., 2018), which was synthesized by Sino US Taihe Biotechnology (Beijing) Co., Ltd. The relative expression levels were calculated by the 2^–△△CT^ method, and analysis was performed in three biological replicates.

**TABLE 2:** Primer sequences used in qRT-PCR.

Gene	Primers sequence (5′-3′)	
	Forward	Reverse
*GA3ox4*	GGTGGGCAAAGGAAATACAAG	AACCCAATCTTTTCAAACTTACCTT
*NIT4*	ACCATCTATTCTGCTGGCTCAT	AACAGGCTGGAGTCTGGAGG
*OPR3*	CGGATTTGCCAAGGAGGTTC	GAGGATCATGATTATTGAGTCGGG
*AAO*	AGAAAACTCCTGAAGAAATCAGGG	TTTACAAGCATTAGAAAAGTCGCAT
*APRT*	CGTGAAATGTGGCAGTGGG	AGCCAGGATTCAATTTTCTGCT
*SRD1*	AGAGGAGAAATGGGTTGTTTA	GTGCACGAAACTCCCCTT
*Ibkn1*	AAAACTCGGGAGATCACTGC	GCAAGCCTCCTCCAATCTC
*Ibkn2*	ACCGGCTTTTCTCGTAGTTG	GGATTCTTGCTGACGTGGAT
*IbMADS1*	CAGGCATATGGCACAAGTGAC	GTTTATTTAACATCAAACACCAA
β*-actin*	AGCAGCATGAAGATTAAGGTTGTAGCAC	TGGAAAATTAGAAGCACTTCCTGTGAAC

### Statistical Analysis

All data were analyzed using SPSS software (version 13.0 for Windows). All data were in triplicates, and the figures were designed by GraphPad Prism software (version 8.4.2 for Windows).

## Results

### Sweetpotato Storage Root Yield Components, Yield, and Appearance Quality

The results of the 2-year field experiment showed that Yan-25 and Pu-32 showed the same effect of phosphorus on storage root components and yield (Table 3). Phosphorus reduced the average weight of storage roots and increased the average number of storage roots per plant and thus yield. The results showed that the highest average storage root number per plant and yield was observed under the P_2_ treatment, which showed a significant difference when compared to the control treatment (*P* < 0.05), while the average weight of the storage root was similar to that of the control treatment. The yield of storage roots increased by 18.99–20.80% in 2020 and by 23.20–25.93% in 2021 under P_2_ treatment. Besides, phosphorus addition decreased the storage root diameter and length, whereas it increased the L/D ratio and CV^–1^ of Yan-25 and Pu-32 cultivars. Compared to the control treatment, the diameter and length of storage root in the P_2_ treatment decreased significantly, while the L/D ratio and CV^–1^ increased significantly (*P* < 0.05). A continuous increase in the phosphorus concentration causes an increase in the length, diameter, and weight of the storage roots and a decrease in the L/D ratio, CV^–1^, average number of storage roots per plant, and yield.

**TABLE 3:** Effect of phosphorus on storage root yield components, yield, and appearance quality.

Y	C	T^†^	Storage root number per plant	Storage root weight (g)	CV^–1^	Storage root diameter (mm)	Storage root length (cm)	L/D ratio	Yield (kg hm^–2^)	Yield increment (%)^‡^
2020	YS-25	P_0_	3.80 d	98.57a	–	–	–	–	23410.71cde	–
		P_1_	3.95cd	97.86a	–	–	–	–	24159.19bcde	3.20
		P_2_	4.70a	96.28a	–	–	–	–	28281.25a	20.80
		P_3_	4.60a	88.30b	–	–	–	–	25385.64b	8.44
		P_4_	4.40abc	86.56b	–	–	–	–	23804.00bc	1.68
	PS-32	P_0_	3.75d	96.36a	–	–	–	–	22585.23de	–
		P_1_	4.05bcd	95.52a	–	–	–	–	24178.50bcd	7.05
		P_2_	4.53ab	94.85a	–	–	–	–	26875.00a	18.99
		P_3_	4.45ab	86.52b	–	–	–	–	24062.50bcde	6.54
		P_4_	4.21abcd	84.44b	–	–	–	–	22219.44e	−1.62
2021	YS-25	P_0_	2.40f	157.18a	1.33	47.26a	13.46a	2.85	23577.65 b	–
		P_1_	2.80d	143.11bc	1.69	42.39b	13.39a	3.16	25043.63b	6.22
		P_2_	3.40a	136.70bcd	2.03	39.54def	12.62a	3.19	29048.25a	23.20
		P_3_	3.20b	124.60d	1.92	38.40f	12.85a	3.34	24919.50b	5.69
		P_4_	2.80d	140.52bc	1.88	41.61bc	13.66a	3.28	24591.63b	4.30
	PS-32	P_0_	2.40f	148.41ab	1.58	41.28bcd	12.82a	3.11	22261.63b	–
		P_1_	2.60e	144.80abc	1.66	41.29bcd	12.41a	3.01	23530.25b	5.70
		P_2_	3.20b	140.17bcd	1.70	39.20ef	12.60a	3.21	28033.13a	25.93
		P_3_	3.00c	132.77cd	2.17	39.67cdef	12.79a	3.22	24894.63b	11.83
		P_4_	2.60e	150.66ab	1.86	40.95bcde	14.39a	3.51	24482.00b	9.97
**ANOVA analysis**										
Y			13517.60**	668.89**	–	–	–	–	11.36	–
C			1.91	0.10	–	19.88*	0.40	–	9.05*	–
T			20.05**	16.63**	–	22.34**	1.26	–	28.61**	–
Y × C			0.16	2.67	–	–	–	–	0.14	–
Y × T			0.67	6.86**	–	–	–	–	0.32	–
C × T			0.31	1.47	–	9.33**	0.44	–	0.15	–
Y × C × T			0.18	1.38	–	–		–	1.13	–

Y, Year; C, Cultivars; T, Treatments. YS-25, Yanshu25; PS-32, Pushu32. Two-way ANOVA, LSD. Values followed by different lowercase letters within a column are significantly different among phosphorus treatments (P < 0.05). *P < 0.05; **P < 0.01. Similarly hereinafter.

*^†^P_0_, 0 kg of P_2_O_5_ hm^–2^; P_1_, 56 kg of P_2_O_5_ hm^–2^; P_2_, 112 kg of P_2_O_5_ hm^–2^; P_3_, 168 kg of P_2_O_5_ hm^–2^; P_4_, 224 kg of P_2_O_5_ hm^–2^.*

*^‡^Compared with the P0 treatment.*

### Storage Root Traits at Canopy Closure Period

At the canopy closure period (40 days after planting), the results obtained after two rounds of field experiment (Table 4) and pot experiment (Table 5) were similar, where Yan-25 and Pu-32 cultivars showed the same effect of phosphorus on storage root traits. In these varieties, the root diameter, average root weight, and the number of storage roots increased initially and then decreased with the increase in phosphorus application rate. Moreover, the average weight and the number of storage roots were the highest under the P_2_ treatment, and there was a significant difference between the P_2_ treatment and the control treatment (*P* < 0.05). The number of storage roots with a diameter above 5 mm was mainly increased.

**TABLE 4:** Effect of phosphorus on storage root traits at canopy closure period under field experiment.

Y	C	T^†^	Storage root diameter (mm)	Storage root number			Avg. storage root weight (g)	Avg. storage root number
				2 < ∅ < 5 mm	5 < ∅ < 10 mm	∅ > 10 mm		
2020	YS-25	P_0_	9.63cd	1.00a	1.00bc	–	3.31de	2.00d
		P_1_	9.80c	1.00a	1.00bc	0.33 b	3.85 c	2.33c
		P_2_	11.03a	1.33a	2.67a	0.33 b	6.65 a	4.33a
		P_3_	10.52b	1.33a	2.00ab	0.67 ab	4.92 b	4.00ab
		P_4_	7.33f	2.00a	1.00bc	–	3.66 cd	3.00b
	PS-32	P_0_	7.60ef	1.00a	1.00bc	–	3.48 cd	2.00d
		P_1_	7.84e	1.33a	1.33bc	1.00 a	3.6 cd	3.67b
		P_2_	9.30d	1.00a	2.67a	0.33 b	5.47 b	4.00ab
		P_3_	6.48g	1.00a	1.00bc	–	2.76 e	2.00d
		P_4_	6.36g	1.33a	0.67c	–	1.88f	2.00d
2021	YS-25	P_0_	10.30de	1.00a	1.33b	0.33a	5.51cd	2.67c
		P_1_	10.66cd	1.00a	1.67ab	0.33a	5.72c	3.00bc
		P_2_	12.33a	0.67a	2.67a	0.67a	7.65a	4.00a
		P_3_	11.32b	0.67a	2.33ab	0.67a	6.45b	3.67ab
		P_4_	9.86e	1.00a	2.00ab	0.33a	5.62cd	3.33b
	PS-32	P_0_	8.86f	1.00a	1.33b	0.33a	4.32e	2.67c
		P_1_	9.24f	1.00a	1.67ab	0.33a	4.85de	3.00bc
		P_2_	10.89bc	0.67a	2.33ab	0.67a	5.96bc	3.67ab
		P_3_	7.86g	1.00a	2.33ab	0.33a	5.41cd	3.67ab
		P_4_	7.05h	1.33a	1.33b	0.33a	4.65e	3.00bc
**ANOVA analysis**								
Y			139.06**	80.82*	7.00	1.32	559.66**	1.33
C			6186.68**	0.04	3.00	0.04	96.61**	1.59
T			244.04**	1.16	9.26**	1.25	130.84**	4.64**
Y × C			0.35	0.89	0.00	0.04	0.25	0.18
Y × T			3.31*	0.36	1.15	0.90	13.01**	0.79
C × T			38.19**	0.15	0.59	0.96	9.05**	1.40
Y × C × T			12.74**	0.53	0.56	0.36	9.12**	1.10

*^†^P_0_, 0 g of P_2_O_5_ kg^–1^; P_1_, 0.02 g of P_2_O_5_ kg^–1^; P_2_, 0.04 g of P_2_O_5_ kg^–1^; P_3_, 0.06 g of P_2_O_5_ kg^–1^; P_4_, 0.08 g of P_2_O_5_ kg^–1^.*

Y, Year; C, Cultivars; T, Treatments. YS-25, Yanshu25; PS-32, Pushu32. Two-way ANOVA, LSD. Values followed by different lowercase letters within a column are significantly different among phosphorus treatments (P < 0.05). *P < 0.05; **P < 0.01.

**TABLE 5:** Effect of phosphorus on storage root traits at canopy closure period under pot experiment.

Y	C	T^†^	Storage root diameter (mm)	Storage root number			Avg. storage root weight (g)	Avg. storage root number
				2 < ∅ < 5 mm	5 < ∅ < 10 mm	∅ > 10 mm		
2019	YS-25	P_0_	6.71b	2.67a	–	0.67a	2.12ab	3.33b
		P_2_	8.46a	1.67a	1.67a	0.67a	2.45a	4.00a
		P_4_	5.94c	2.00a	–	1.00a	2.02abc	3.00b
	PS-32	P_0_	5.78c	1.67a	–	1.33a	1.59c	3.00b
		P_2_	6.26bc	2.00a	0.67b	1.00a	1.97bc	3.67ab
		P_4_	4.95d	1.67a	–	1.33a	1.10d	3.00b
**ANOVA analysis**								
C			197.87**	3.00	0.00**	4.00	182.59**	0.31
T			76.87**	0.42	24.50**	0.27	10.21**	1.68
C × T			13.16**	1.26	4.50*	0.09	1.21f	0.08
2020	YS-25	P_0_	5.02f	1.33ab	1.33ab	1.00b	2.17de	3.67c
		P_1_	7.79b	1.00b	1.00ab	2.00a	2.42cd	4.00b
		P_2_	8.86a	1.67ab	1.67ab	1.00b	3.05a	4.67a
		P_3_	7.23c	1.67ab	1.33ab	1.00b	2.56bc	4.00b
		P_4_	5.66e	1.00b	0.67b	1.67ab	2.41cd	3.33d
	PS-32	P_0_	2.99h	3.00a	0.67b	–	1.13	3.67c
		P_1_	6.10d	0.33b	3.00a	0.67bc	2.61bc	4.00b
		P_2_	7.33c	1.00b	3.00a	0.33c	2.92ab	4.33ab
		P_3_	6.23d	1.00b	2.00ab	1.33ab	2.34cd	4.33ab
		P_4_	3.92g	1.00b	2.00ab	0.33c	1.96e	3.33d
**ANOVA analysis**								
C			6384.01**	0.04	6.04	6.86	51.05*	1.00
T			907.72**	2.07	1.12	1.07	31.22**	1.78
C × T			11.71**	1.70	1.08	1.07	6.86**	0.15

*^†^P_0_, 0 g of P_2_O_5_ kg^–1^; P_1_, 0.02 g of P_2_O_5_ kg^–1^; P_2_, 0.04 g of P_2_O_5_ kg^–1^; P_3_, 0.06 g of P_2_O_5_ kg^–1^; P_4_, 0.08 g of P_2_O_5_ kg^–1^.*

C, Cultivars; T, Treatments. YS-25, Yanshu25; PS-32, Pushu32. Two-way ANOVA, LSD. Values followed by different lowercase letters within a column are significantly different among phosphorus treatments (P < 0.05). *P < 0.05; **P < 0.01.

### Root Traits During Storage Root Initiation and Formation

**FIGURE 1:**
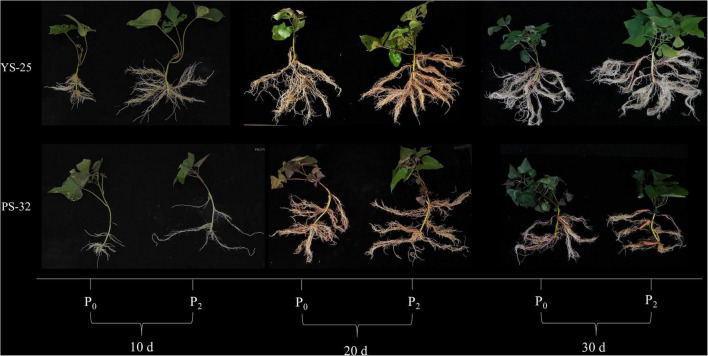
Effect of phosphorus on sweetpotato root growth and development during storage root formation. ^†^YS-25, Yanshu25; PS-32, Pushu32. ^‡^P_0_, 0 g of P_2_O_5_ kg^–1^; P_2_, 0.04 g of P_2_O_5_ kg^–1^.

Figure 1 shows that compared to the control treatment, the P_2_ treatment promoted the root development of sweetpotato, and the detailed results are summarized as follows.

During storage initiation (10–20 days after planting), the total root length, root surface area, root diameter, root volume, and root tip number first increased and then decreased with the increment of phosphorus concentration in Yan-25 and Pu-32 cultivars. Moreover, the total root length, root surface area, diameter, root volume, and root tip number were the highest after P_2_ treatment, and these traits showed significant differences when compared to the control treatment (*P* < 0.05) (Table 6).

**TABLE 6:** Effect of phosphorus on total root length, root surface area, root diameter, root volume, and root tip number during storage root initiation.

DAP (d)	C	T^†^	Root total length (cm)	Root surface area (cm^2^)	Root diameter (mm)	Root volume (cm^3^)	Root tips number
10	YS-25	P_0_	1022.14b	214.72b	0.69a	3.92b	1273.67bc
		P_2_	1496.29a	345.33a	0.69a	6.05a	1568.33a
		P_4_	972.14bc	196.94bc	0.66a	3.19cd	1139.33c
	PS-32	P_0_	803.56bc	164.56bc	0.65a	2.69de	1358.33bc
		P_2_	854.26bc	184.53bc	0.69a	3.53bc	1422.67ab
		P_4_	782.46c	156.67c	0.64a	2.51be	984.33d
**ANOVA analysis**							
C			52.98*	154.68**	0.15	189.92**	0.12
T			33.06**	12.48**	0.04	75.62**	12.58**
C × T			20.01**	6.17*	0.01	16.38**	1.24
20	YS-25	P_0_	2568.22ab	522.89d	0.64b	9.78d	2443.00b
		P_2_	3051.07a	783.81a	0.66b	10.88c	2702.00ab
		P_4_	2898.22ab	568.73c	0.62b	8.90e	2655.00ab
	PS-32	P_0_	2688.56ab	672.44b	1.17ab	12.80b	2856.67ab
		P_2_	2987.30ab	740.13a	1.32a	14.91a	3085.00a
		P_4_	2443.65b	480.81d	1.04ab	7.53f	2650.67ab
**ANOVA analysis**							
C			3.77	0.25	21.02*	261.59**	58.55**
T			2.32	172.23**	1.13	262.72**	1.64
C × T			1.13	46.51**	0.63	95.71**	1.14

*DAP, Days after planting.*

*^†^P_0_, 0 g of P_2_O_5_ kg^–1^; P_1_, 0.02 g of P_2_O_5_ kg^–1^; P_2_, 0.04 g of P_2_O_5_ kg^–1^; P_3_, 0.06 g of P_2_O_5_ kg^–1^; P_4_, 0.08 g of P_2_O_5_ kg^–1^.*

C, Cultivars; T, Treatments. YS-25, Yanshu25; PS-32, Pushu32. Two-way ANOVA, LSD. Values followed by different lowercase letters within a column are significantly different among phosphorus treatments (P < 0.05). *P < 0.05; **P < 0.01.

**FIGURE 2:**
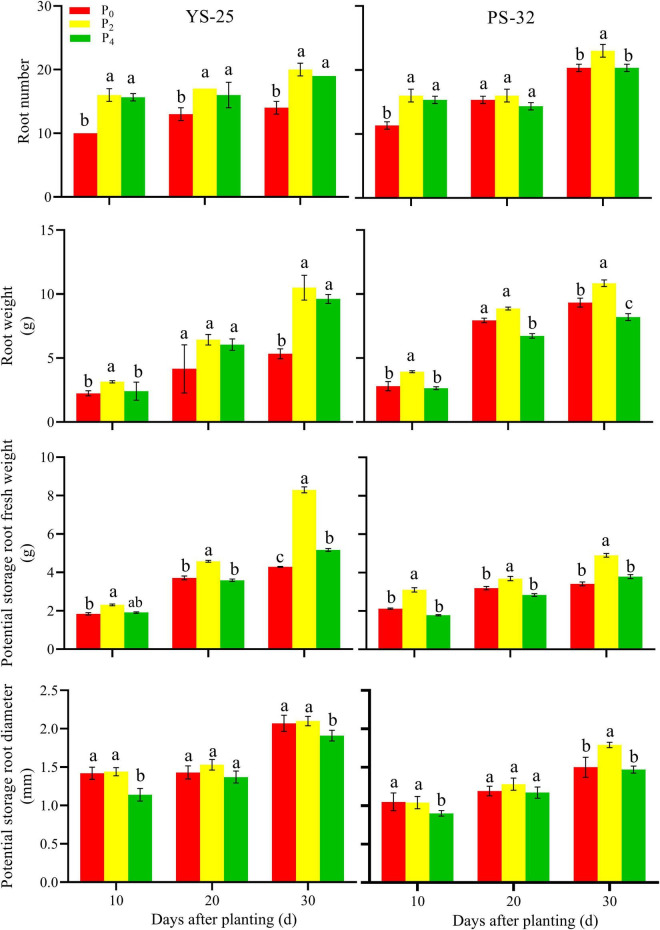
Effect of phosphorus on root number, root weight, potential storage root fresh weight, and potential storage root diameter during storage formation (2019). ^†^YS-25, Yanshu25; PS-32, Pushu32. ^‡^P_0_, 0 g of P_2_O_5_ kg^–1^; P_2_, 0.04 g of P_2_O_5_ kg^–1^; P_4_, 0.08 g of P_2_O_5_ kg^–1^. ^§^ Error bars represent 1 SD (*n* = 3) within the same column, and different letters (a and b) indicate significant differences between N treatments (*P* < 0.05).

**FIGURE 3:**
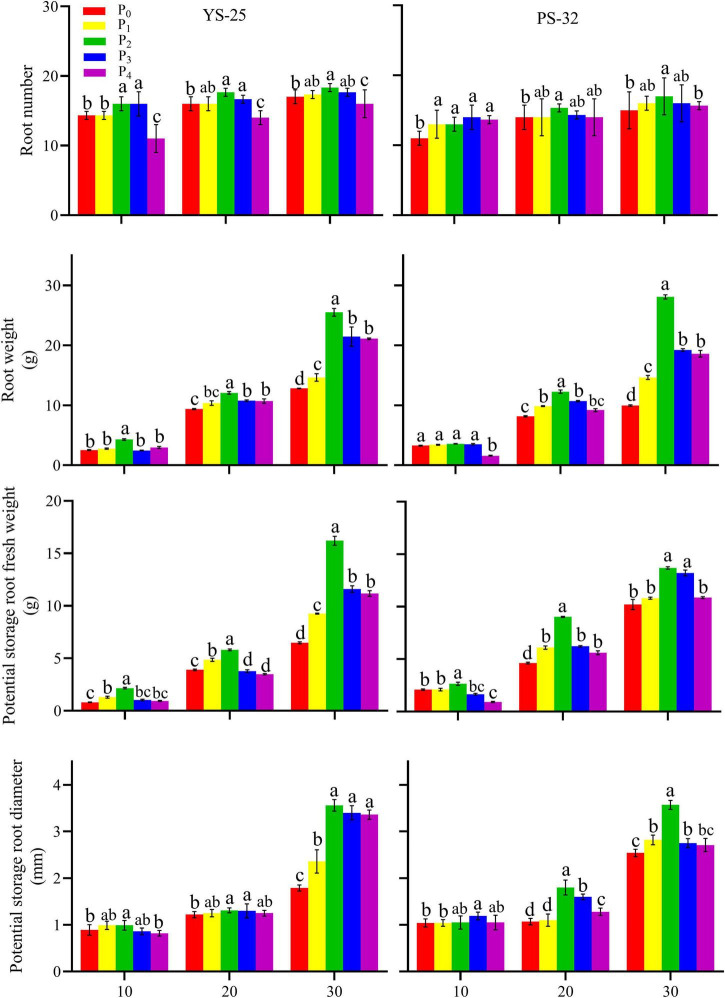
Effect of phosphorus on root number, root weight, potential storage root fresh weight, and potential storage root diameter during storage formation (2020). ^†^YS-25, Yanshu25; PS-32, Pushu32. ^‡^P_0_, 0 g of P_2_O_5_ kg^–1^; P_1_, 0.02 g of P_2_O_5_ kg^–1^; P_2_, 0.04 g of P_2_O_5_ kg^–1^; P_3_, 0.06 g of P_2_O_5_ kg^–1^; P_4_, 0.08 g of P_2_O_5_ kg^–1^. ^§^ Error bars represent 1 SD (*n* = 3) within the same column, and different letters (a and b) indicate significant differences between N treatments (*P* < 0.05).

During the storage root formation (0–30 days after planting), the root number, root weight, potential storage root diameter, and potential storage root weight increased first and then decreased with the increase of phosphorus application in Yan-25 and Pu-32. The highest root number, root weight, potential storage root diameter, and potential storage root weight were observed under the P_2_ treatment, and these traits showed significant differences when compared to the control treatment (*P* < 0.05) (Figures 2, 3).

### Endogenous Hormones Concentration in the Root During Storage Root Formation

**FIGURE 4:**
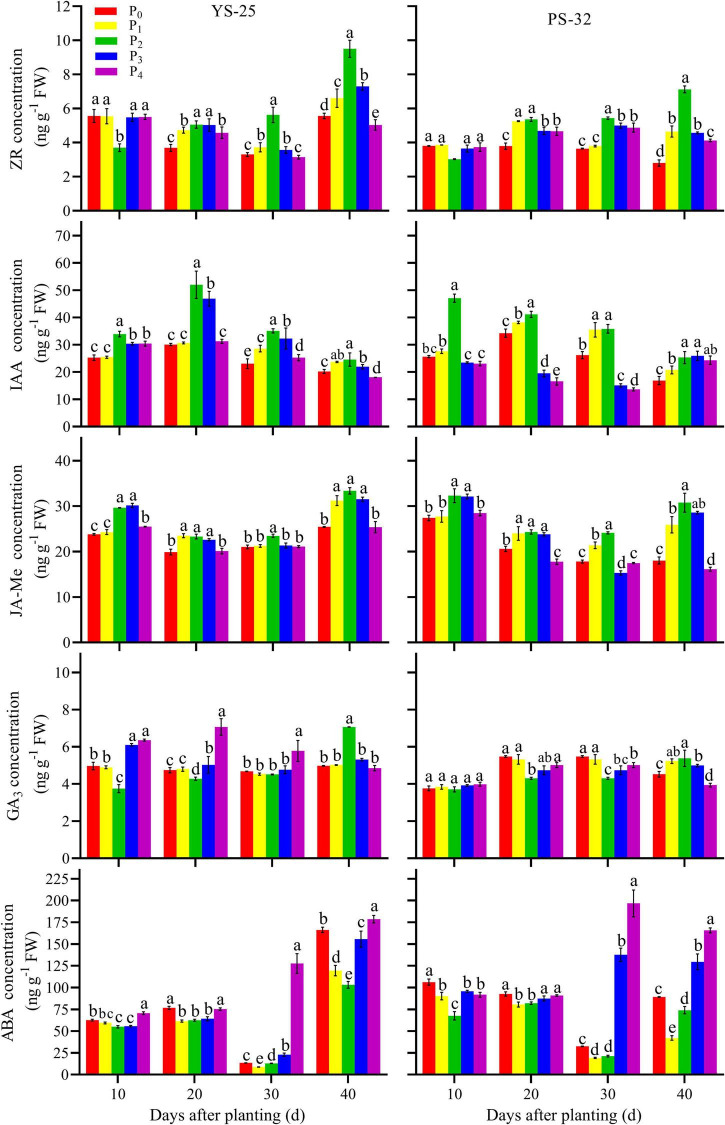
Effect of phosphorus on endogenous phytohormone concentration in root during storage root formation. ^†^YS-25, Yanshu25; PS-32, Pushu32. ^‡^P_0_, 0 g of P_2_O_5_ kg^–1^; P_1_, 0.02 g of P_2_O_5_ kg^–1^; P_2_, 0.04 g of P_2_O_5_ kg^–1^; P_3_, 0.06 g of P_2_O_5_ kg^–1^; P_4_, 0.08 g of P_2_O_5_ kg^–1^. ^§^ Error bars represent 1 SD (*n* = 3) within the same column, and different letters (a and b) indicate significant differences between N treatments (*P* < 0.05).

Yan-25 and Pu-32 had the same effect of phosphorus on endogenous hormones concentration (Figure 4). After 10 days of planting, the ZR concentration in young roots first decreased and then increased with the increase of phosphorus concentration, and the effect was significantly different from that of the control treatment (*P* < 0.05). After 20 and 40 days of planting, the ZR concentration increased at first and then decreased in young roots. Moreover, the ZR concentration was the highest under the P_2_ treatment and was significantly different from that of the control treatment (*P* < 0.05). After 0–40 days of planting, the concentrations of IAA and JA-Me first increased and then decreased with the increase of phosphorus in young roots. Moreover, the levels of these compounds were the highest in the P_2_ treatment and were significantly different from those of the control treatment (*P* < 0.05). After 30 days of planting, the GA_3_ concentration decreased first and then increased with the increase of phosphorus in young roots. Furthermore, the GA_3_ concentration was found to be lowest under the P_2_ treatment, and the GA_3_ concentration in P_2_ treatment was significantly different from that of the control treatment at 20 and 30 days after planting. After 40 days of planting, the GA_3_ concentration increased first and then decreased with the increment of phosphorus application in young roots. Moreover, the GA_3_ concentration was found to be the highest under the P_2_ treatment and showed a significant difference from that of the control treatment (*P* < 0.05). After 40 days of planting, the ABA concentration decreased first and then increased with the increment of phosphorus application in young roots. Furthermore, the ABA concentration under the P_2_ treatment was found to be the lowest and showed a significant difference from that of the control treatment (*P* < 0.05).

### Gene Expression in Roots During Storage Root Formation

**FIGURE 5:**
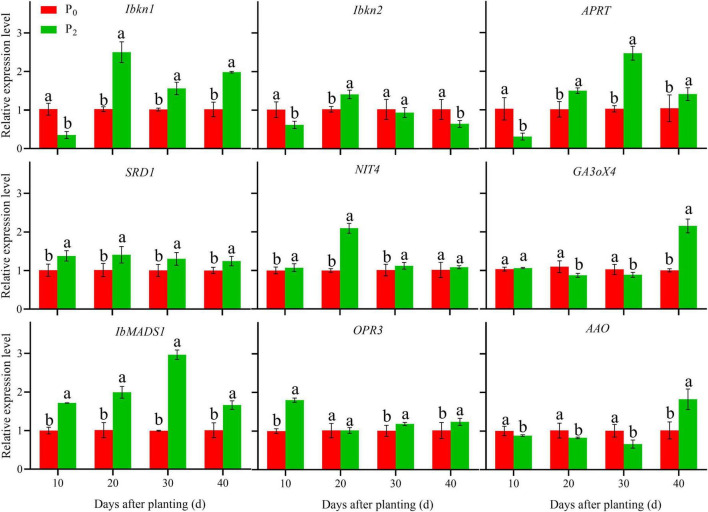
Effect of phosphorus on gene expression in YS-25 root during storage root formation. ^†^YS-25, Yanshu25. ^‡^P_0_, 0 g of P_2_O_5_ kg^–1^; P_2_, 0.04 g of P_2_O_5_ kg^–1^. ^§^ Error bars represent 1 SD (*n* = 3) within the same column, and different letters (a and b) indicate significant differences between N treatments (*P* < 0.05).

**FIGURE 6:**
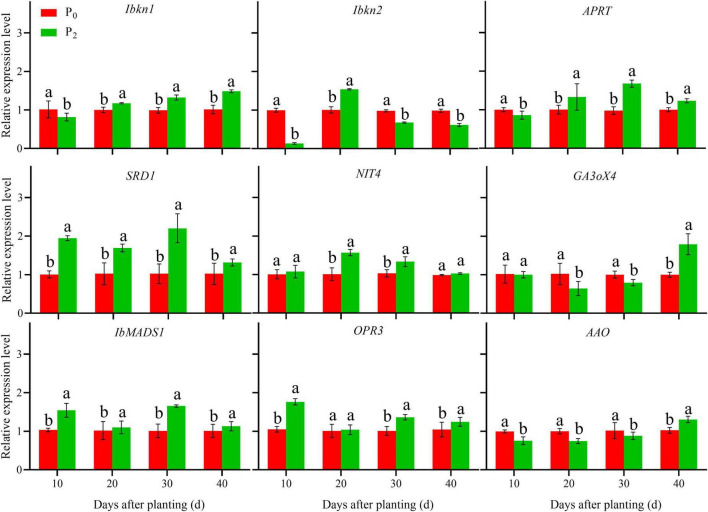
Effect of phosphorus on gene expression in PS-32 root during storage root formation. ^†^PS-32, Pushu32. ^‡^P_0_, 0 g of P_2_O_5_ kg^–1^; P_2_, 0.04 g of P_2_O_5_ kg^–1^. ^§^ Error bars represent 1 SD (*n* = 3) within the same column, and different letters (a and b) indicate significant differences between N treatments (*P* < 0.05).

During storage root formation (0–40 days after planting), the effect of phosphorus on the gene expression of YS-25 and PS-32 in potential storage roots was similar (Figures 5, 6). Compared to the control treatment, the expression of *Ibkn1*, *Ibkn2*, and *APRT* in the potential storage roots of both the cultivars treated with P_2_ decreased significantly at 10 days after planting, whereas the expression of these genes in the potential storage roots of both cultivars increased significantly at 20 days after planting. After 30 days of planting, the expression of *Ibkn1* and *APRT* in the potential storage roots of both the cultivars increased significantly, while the expression of *Ibkn2* decreased (*P* < 0.05). Compared to the control treatment, the expression of *SRD1*, *NIT4*, *IbMADS1*, and *OPR3* genes in the potential storage roots of both the cultivars treated with P_2_ increased significantly after 40 days of planting. However, decrease in expression of *NIT4* in the PS-32 potential storage roots on the 10th day, *OPR3* in the YS-25 and PS-32 roots on the 20th day, and *NIT4* in the YS-25 and PS-32 roots on the 40th day after the planting process was observed. Compared to the control treatment, the expression of *GA3oX4* in potential storage roots treated with P_2_ showed no significant difference at 10 days of planting, but decreased significantly from 20 to 30 days of planting and increased significantly after 40 days of planting. P_2_ treatment showed a significant decrease in the expression of *AAO* from 10 to 30 days after planting in comparison to the control treatment and increased significantly at 40 days after planting, with the difference reaching a significant level (*P* < 0.05).

## Discussion

### Effect of Phosphorus on Endogenous Hormone Metabolism in Potential Storage Roots

The adenine phosphoribosyltransferase (APRT) and its gene *APRT* participate in the activation of cytokinin biosynthesis in plants (Mok and Mok, 2001; Auer, 2002; Zhou et al., 2006; Wu et al., 2008). *Nitrilase 4 (NIT4)* exhibits nitrilase activity and catalyzes the conversion of indole acetonitrile to IAA (Piotrowski et al., 2001; Wang et al., 2019). 12-oxo-10,15(Z)-phytodienoic acid reductase 3 (OPR3) is a key enzyme that is involved in the JA synthesis (Schaller and Stintzi, 2009; Wasternack and Hause, 2013). Plants synthesize ABA using the carotenoid biosynthesis pathway initiated by β-carotene, and in this pathway, abscisic aldehyde oxidase (AAO) converts xanthoxin into ABA (Chen et al., 2020). GA3-oxidase (GA3ox) is a key enzyme in gibberellin (GA) biosynthesis (Li and Li, 2019).

During the storage root formation, there was no difference in the concentration of ABA in the fibrous root (diameter below 2 mm) and the thickness of the roots (Nakatani and Komeichi, 1991). Moreover, the JA (10–20 μM) combined with BA (10 μM) could induce the expression of *IbMADS1* and promote storage root formation under *in vitro* culture conditions (Ku, 2008). The exogenous application of GA_3_ could delay storage root formation and reduce the number of storage roots (Singh et al., 2019). Furthermore, ammonium nitrogen (ammonium sulfate) could regulate the expression of *SRD1*, leading to a change in the IAA concentration, and the expression of *Ibkn1* and *Ibkn2*, leading to a change in the ZR concentration, thus increasing the number of storage roots per plant and yield (Si et al., 2018). These findings are similar to the results of phosphorus treatment obtained in the present study.

The results of our studies showed that an adequate level of phosphorus could significantly decrease the expression of *Ibkn1*, *Ibkn2*, and *APRT* genes, and also the concentration of ZR in the young roots after 10 days of planting at the stage of storage root formation. Similarly, it could increase the expression of *Ibkn1* and *APRT* significantly, and the concentration of ZR in the young roots after 20–40 days of planting. Furthermore, it increased the expression of *SRD1*, *NIT4*, *IbMADS1*, and *OPR3* in the young roots from 10 to 40 days after planting, and also increased the concentrations of IAA and JA. It significantly decreased *GA3oX4* expression and GA3 concentration in the young roots at 20–30 days after planting, and significantly increased *GA3oX4* expression and GA3 concentration in the young roots after 40 days of planting. Similarly, it significantly decreased *AAO* expression and ABA concentration in young roots at 10–30 days after planting, and significantly increased *AAO* expression and ABA concentration in the young roots after 40 days of planting.

The effect of different phosphorus (P) concentrations on the growth and physiological characteristics of maize and rice have been identified, which revealed that P starvation led to an increase in the concentration of IAA and GA_3_, and no significant change was observed in the concentration of ABA (Shen et al., 2012; Ding et al., 2021). It is suggested that the effects of P concentrations on the growth of maize and rice may be associated with the changes in endogenous hormones. Our study results confirm that this scientific phenomenon also exists in sweetpotato and that phosphorus could regulate the metabolism of endogenous hormones in the potential storage roots during storage root formation.

### Effect of Phosphorus on Storage Root Formation and Yield

The application of adequate concentrations of phosphorus could improve P utilization efficiency (Wang et al., 2017), yield, and product yield (El-Deen et al., 2011). It also improves the length and nutritional quality, such as starch and sugar content, of the storage roots (El-Deen et al., 2011; Villordon et al., 2018). In our previous studies, we found that appropriate phosphorus application could increase the number of storage roots per plant, average weight of storage roots, and the total weight of storage roots per plant during the canopy closure period (Liang et al., 2021). The current study showed that during storage root formation, total root length, root surface area, root diameter, root volume, root tip number, root number, root weight, potential storage root diameter, and potential storage root weight first increased and then decreased with the increment of phosphorus application in sweetpotato. Furthermore, the highest increment in these parameters was observed under P_2_ treatment. At the canopy closure period (after 40 days of planting), the root diameter, average root weight, and average root number increased first and then decreased with the increment of phosphorus application in sweetpotato. Moreover, the average root weight and root number were found to be the highest (the number of roots with a diameter above 5 mm increased significantly) under P_2_ treatment. Phosphorus application decreased the average root weight of sweetpotato (the average root weight under P_2_ treatment was similar to that of the control) but increased the average root number per plant and root yield (maximum increase was observed under P_2_ treatment). Phosphorus application, particularly P_2_ treatment, significantly decreased the diameter, length, and average weight of storage roots, and increased L/D ratio, CV^–1^, average quantity of storage roots per plant, and yield, when compared to the control treatment. Continuous increase in the level of phosphorus enhances the diameter, length, and average weight of storage root, and decreases the L/D ratio, CV^–1^, the average number of storage roots per plant, and yield of storage root. Thus, proper application of phosphorus fertilized could promote root growth and development, conducive to storage root formation, increase the storage root diameter, average weight of storage root, and the number of storage roots at canopy closure period, which further contributed to the increase of yield (obtain the highest yield) and best appearance quality at harvest.

## Conclusion

In this work, the possible response mechanisms of storage root formation, yield, and appearance quality to phosphorus in sweetpotato were explained by the concentration of endogenous hormones and the expression of related regulatory genes in potential storage root. Hence, it could be suggested that phosphorus is a limiting factor to endogenous hormone metabolism in potential storage root formation, yield, and appearance quality in sweetpotato. Present research can provide theoretical support for phosphorus management in sweetpotato to obtain high yield and quality. Since in this study, the results of endogenous hormone metabolism were obtained from the storage root formation period, further study in the storage root bulking period (from 40 to 120 days after planting) is recommended in order to confirm these findings.

## Data Availability Statement

The original contributions presented in the study are included in the article/Supplementary Material, further inquiries can be directed to the corresponding author/s.

## Author Contributions

C-CS and H-JL conceived the study. C-CS and G-PZ designed the experimental procedures. C-CS, Q-GL, NW, and Y-LC carried out the experimental work. C-CS wrote the manuscript. SK polished the language and reviewed the manuscript. All authors have read and agreed to the published version of the manuscript.

## Conflict of Interest

The authors declare that the research was conducted in the absence of any commercial or financial relationships that could be construed as a potential conflict of interest.

## Publisher’s Note

All claims expressed in this article are solely those of the authors and do not necessarily represent those of their affiliated organizations, or those of the publisher, the editors and the reviewers. Any product that may be evaluated in this article, or claim that may be made by its manufacturer, is not guaranteed or endorsed by the publisher.
