# Supplementary figures and images for: Response Mechanism of Endogenous Hormones of Potential Storage Root to Phosphorus and Its Relationship With Yield and Appearance Quality of Sweetpotato
Source: Front Plant Sci. 2022 May 23;13:872422. doi: 10.3389/fpls.2022.872422 (PMC9168888; doi:10.3389/fpls.2022.872422)

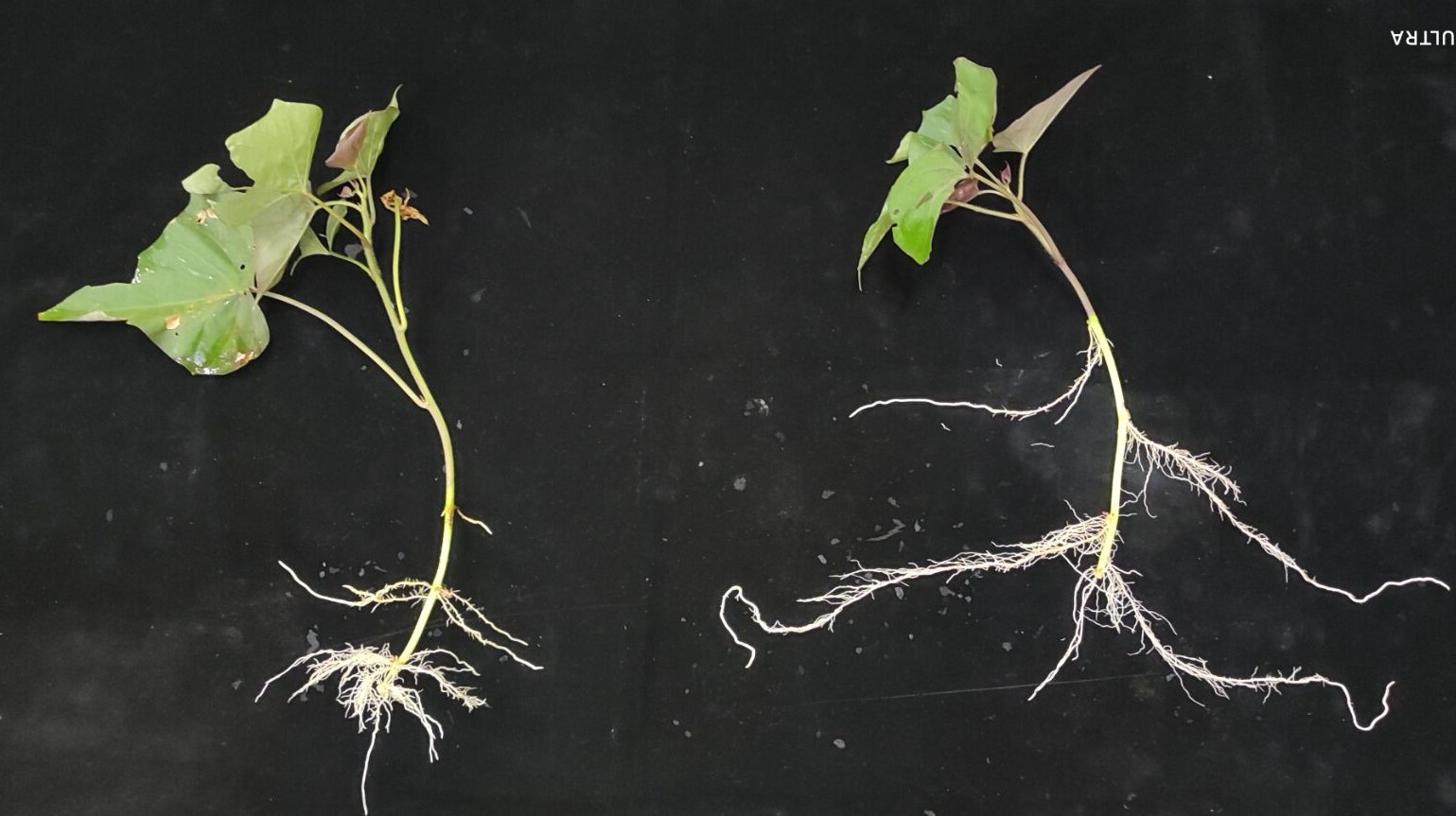

Supplement: Supplementary file 1 [file Data_Sheet_1.ZIP › photos/PS-32 10d.jpg]

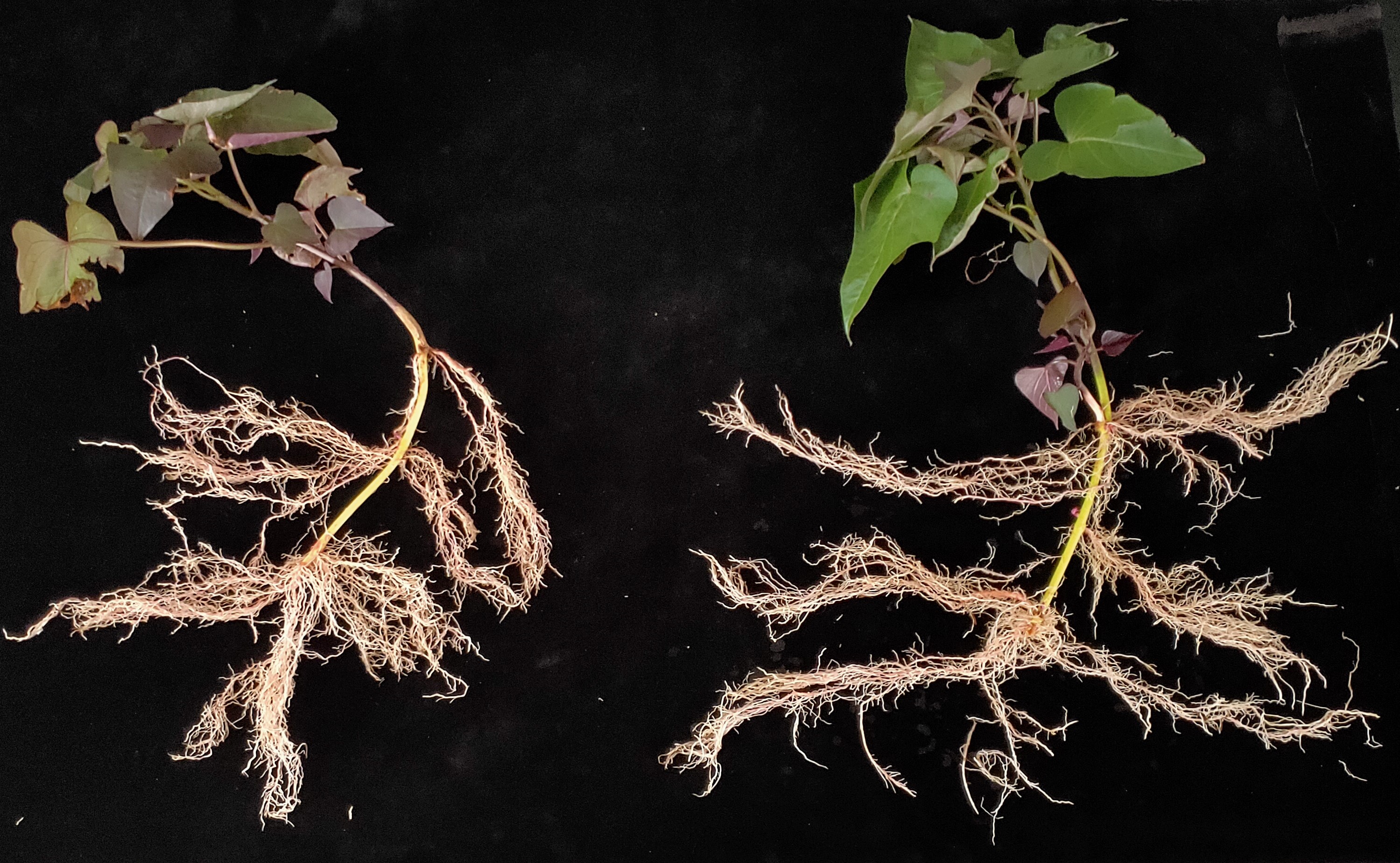

Supplement: Supplementary file 1 [file Data_Sheet_1.ZIP › photos/PS-32 20d.jpg]

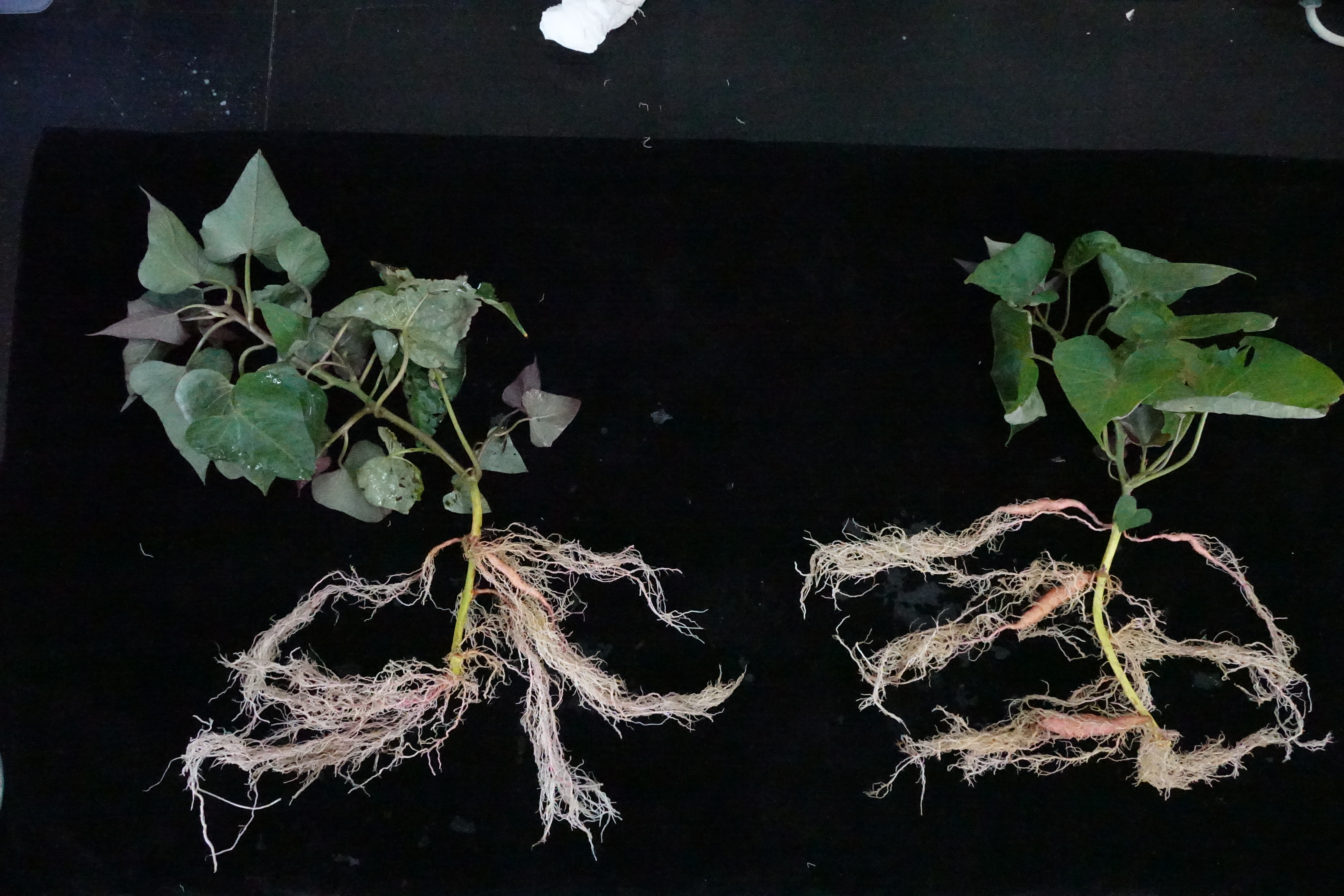

Supplement: Supplementary file 1 [file Data_Sheet_1.ZIP › photos/PS-32 30d.jpg]

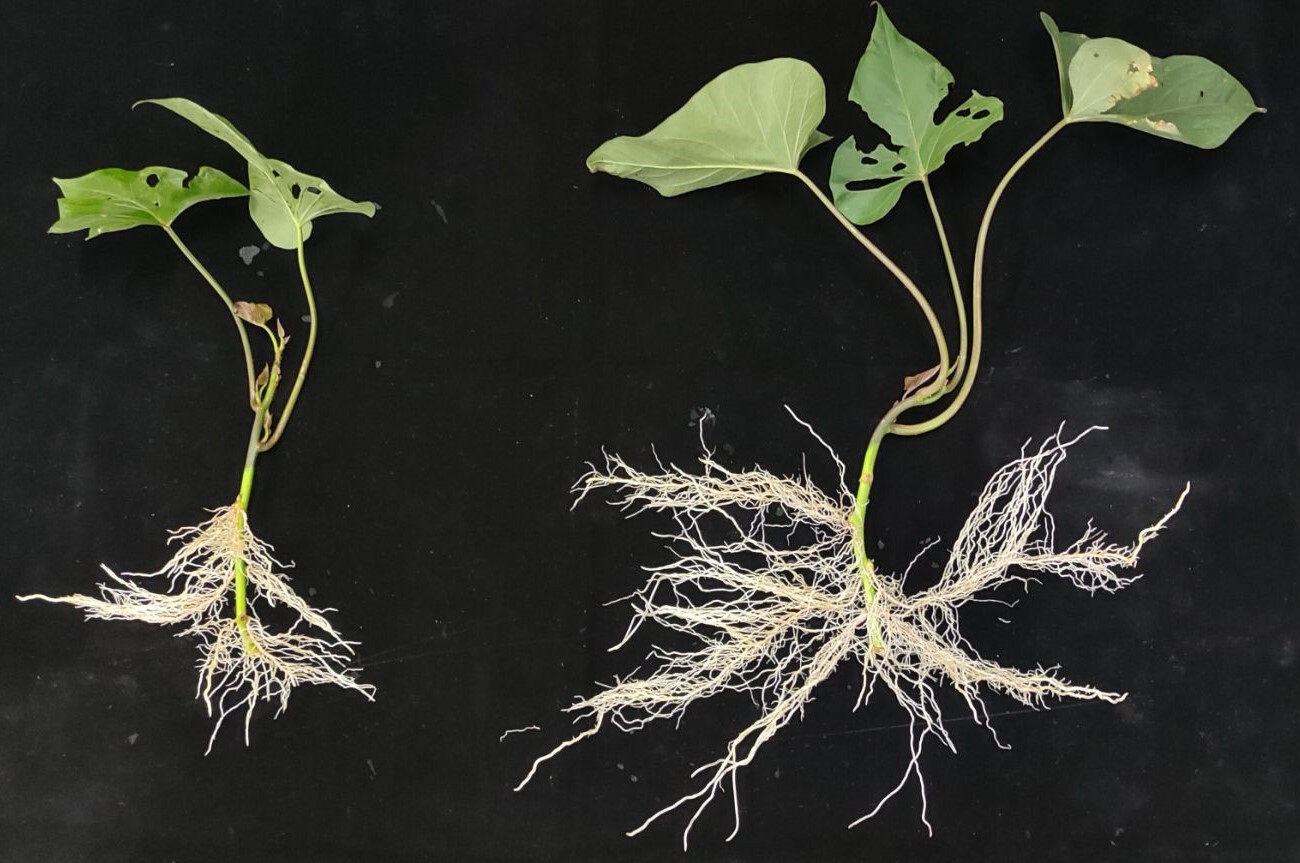

Supplement: Supplementary file 1 [file Data_Sheet_1.ZIP › photos/YS-25 10d.jpg]

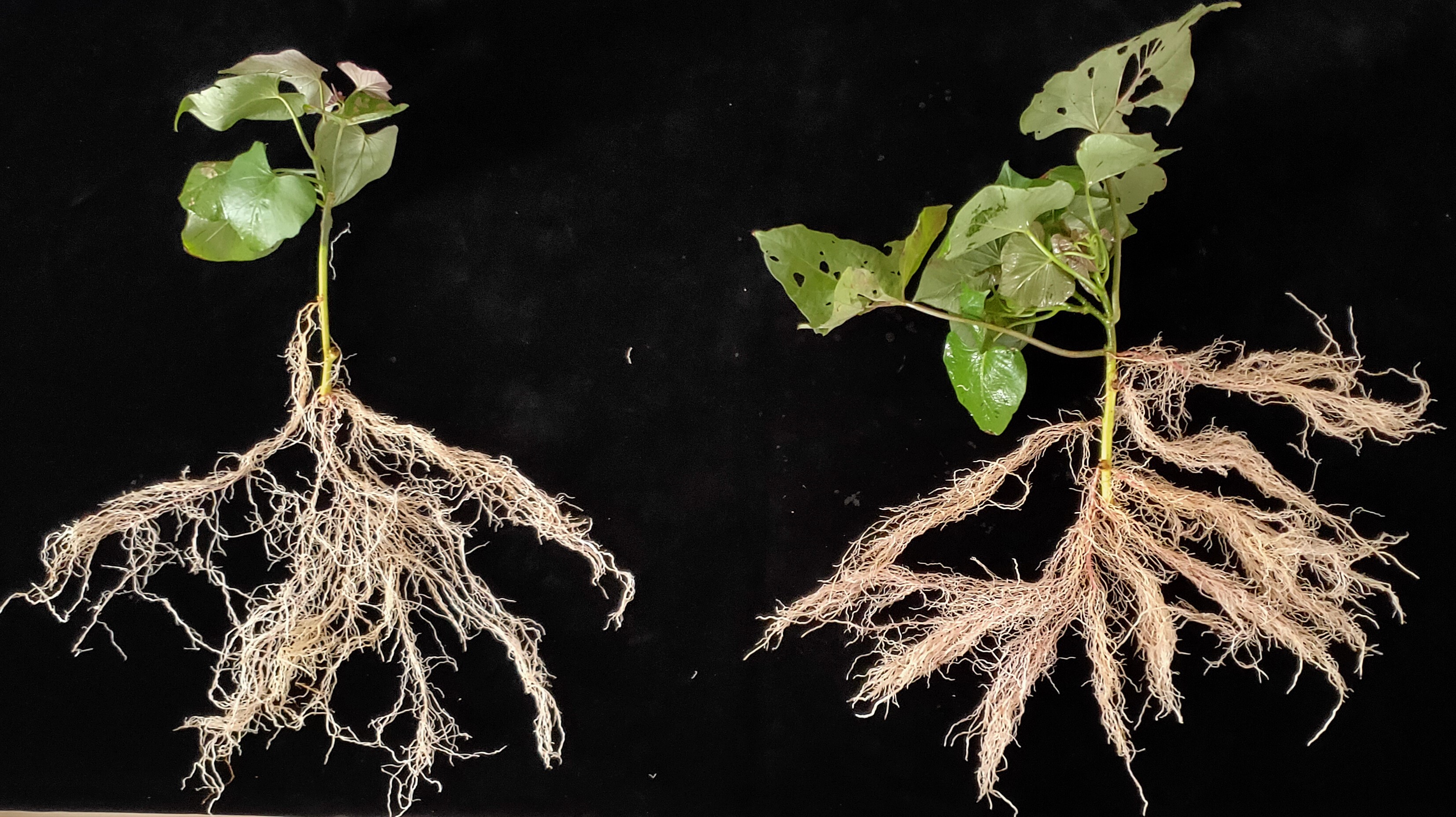

Supplement: Supplementary file 1 [file Data_Sheet_1.ZIP › photos/YS-25 20d.jpg]

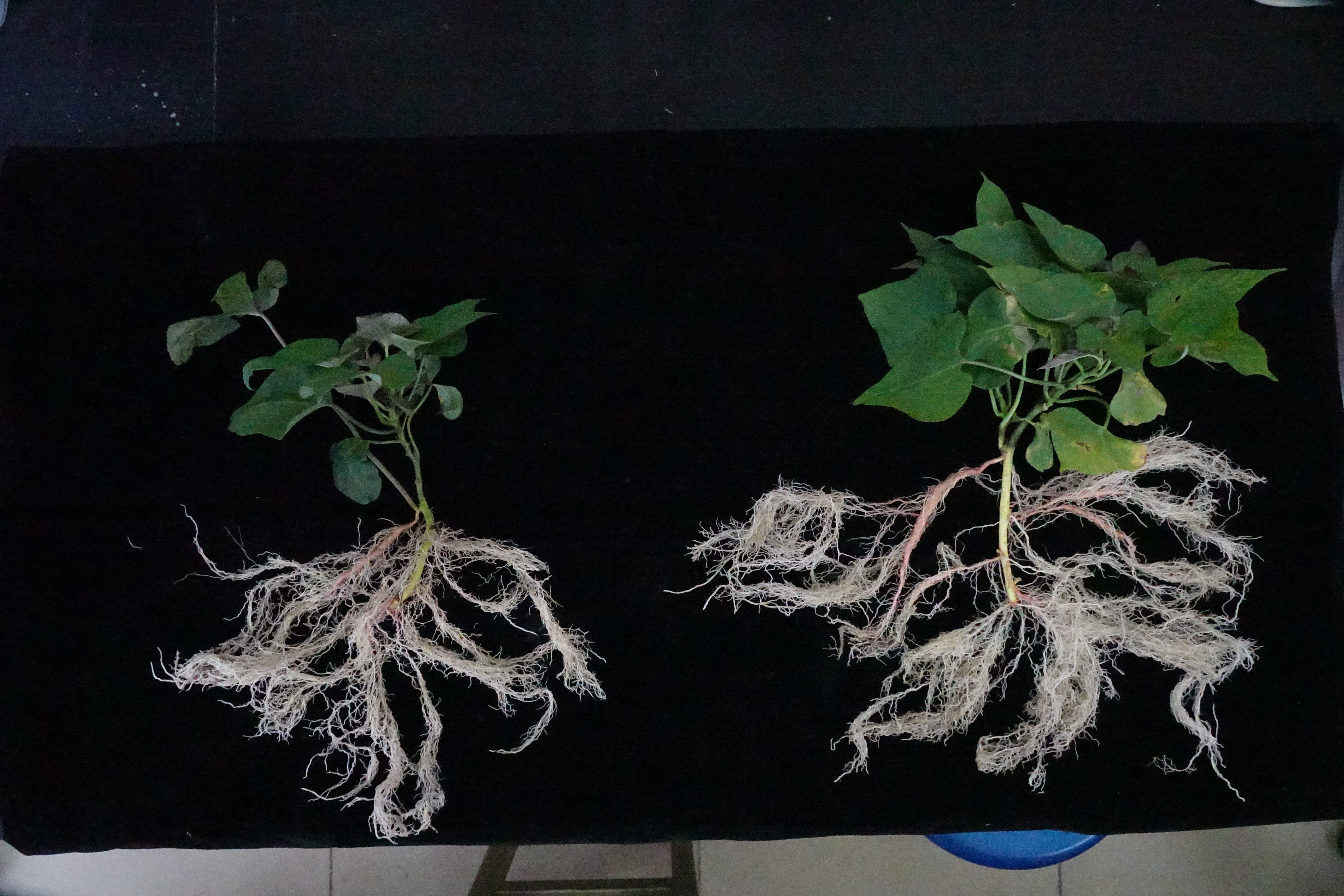

Supplement: Supplementary file 1 [file Data_Sheet_1.ZIP › photos/YS-25 30d.jpg]
